# Application of Raman Spectroscopic Imaging to Assess the Structural Changes at Cell-Scaffold Interface

**DOI:** 10.3390/ijms22020485

**Published:** 2021-01-06

**Authors:** Grzegorz Kalisz, Agata Przekora, Paulina Kazimierczak, Barbara Gieroba, Michal Jedrek, Wojciech Grudzinski, Wieslaw I. Gruszecki, Grazyna Ginalska, Anna Sroka-Bartnicka

**Affiliations:** 1 Department of Biopharmacy, Medical University of Lublin, Chodzki 4a, 20-093 Lublin, Poland; grkalisz@gmail.com (G.K.); barbaragieroba@umlub.pl (B.G.); michaljanjedrek@gmail.com (M.J.); 2 Department of Biochemistry and Biotechnology, Medical University of Lublin, Chodzki 1, 20-093 Lublin, Poland; paulina.kazimierczak@umlub.pl (P.K.); g.ginalska@umlub.pl (G.G.); 3 Collegium Medicum, Cardinal Stefan Wyszynski University in Warsaw, Dewajtis 5, 01-815 Warsaw, Poland; 4 Department of Biophysics, Institute of Physics, Maria Curie-Sklodowska University, pl. Marii Curie-Sklodowskiej 1, 20-031 Lublin, Poland; wojciech.grudzinski@poczta.umcs.lublin.pl (W.G.); wieslaw.gruszecki@poczta.umcs.lublin.pl (W.I.G.); 5 Department of Genetics and Microbiology, Institute of Microbiology and Biotechnology, Maria Curie-Sklodowska University, Akademicka 19, 20-033 Lublin, Poland

**Keywords:** Raman spectroscopy, chemical imaging, hydroxyapatite composite, scaffold interface, tissue engineering

## Abstract

Raman spectroscopic imaging and mapping were applied to characterise three-compound ceramic composite biomaterial consisting of chitosan, β-1,3-d-glucan (curdlan) and hydroxyapatite (HA) developed as a bone tissue engineering product (TEP). In this rapidly advancing domain of medical science, the urge for quick, reliable and specific method for products evaluation and tissue–implant interaction, in this case bone formation process, is constantly present. Two types of stem cells, adipose-derived stem cells (ADSCs) and bone marrow-derived stem cells (BMDSCs), were cultured on composite surface. Raman spectroscopic imaging provided advantageous information on molecular differences and spatial distribution of compounds within and between the cell-seeded and untreated samples at a microscopic level. With the use of this, it was possible to confirm composite biocompatibility and bioactivity in vitro. Deposition of HA and changes in its crystallinity along with protein adsorption proved new bone tissue formation in both mesenchymal stem cell samples, where the cells proliferated, differentiated and produced biomineralised extracellular matrix (ECM). The usefulness of spectroscopic Raman imaging was confirmed in tissue engineering in terms of both the organic and inorganic components considering composite–cells interaction.

## 1. Introduction

Modern methods for bone lesion treatment are developing in a wide range of approaches, with meticulous analysis of biological and physical properties of connective tissue. Injuries, defects or tissue loss significantly diminishes patient’s quality of life, causing pain or restriction of movement during recovery and rehabilitation period. Extensive bone tissue defects caused by trauma, infection or neoplastic resection as well as hindered regeneration due to radiotherapy, necrosis or osteoporosis create demand for bone substitute materials as grafts in surgeries [1]. The global bone substitutes market is expected to reach almost 3.9 billion USD by 2026 according to Transparency Market Research Report from 2018 [2]. Autologous bone grafts are considered to be a close future gold standard for reconstructive applications in the biomedical sciences. Nowadays, this approach encounters issues associated with limited availability of autografts, chronic pain, morbidities and infections [3]. The solution for drawbacks of investigation process might be streamlined development of tissue engineering process involving stiff, inorganic matter with autologous cell transplant, minimising a lot of adverse reactions by biomimetic properties of the product [4]. In tissue engineered bone grafts, there are two or three main components forming implant: biocompatible porous scaffold and progenitor cells and/or growth factors/cytokines [5]. MSCs (mesenchymal stem cells) are able to efficiently differentiate along an osteogenic lineage, while occurring in multiple tissues and considered ethically acceptable. Multipotency and autonomous growth make MSCs promising candidates for use in bone tissue restoration [6]. Many studies have demonstrated that, in the case of in vivo implantations, only a small fraction of cells actually differentiate into bone tissue. Particularly for MSCs, the majority of the improvement in bone repair appears to arise from their paracrine functions (e.g., secreting trophic and angiogenic growth factors) that then promote the settling of the bone defect by endogenous cells that then initiate repair. The role of transplanted MSCs in bone repair therefore is mainly a supportive one in angiogenesis, immune modulation, and cell recruitment rather than directly participating in the repair [7,8,9]. The most studied and characterised mesenchymal stem cells are bone marrow derived stem cells, but their autologous harvesting is an invasive procedure with potential complications and limited amount of aspirate, containing only up to 0.01% of stem cells [10]. Despite the restrictions on obtaining, the BMDSCs (bone marrow-derived stem cells) are preferentially selected for supported bone regeneration studies [11]. Recent reports have indicated that mesenchymal stem cells acquired from adipose tissue have comparable morphology, phenotype and differentiation potential to BMDSCs. ADSCs (adipose-derived stem cells) seem to be promising source of cells for applications in tissue engineered biomaterials [10]. It was reported that both ADSCs and BMDSCs have the ability to proliferate and differentiate on the biomaterial scaffold, however the process is influenced by multiple features of the scaffold [3]. Microstructure and chemical composition seem to play an important role in proliferation control. Macroporous structure of composite and additional components stimulating cell adhesion are considered as crucial at early stage of graft integration. Structure based on hydroxyapatite (HA) and chitosan (CHIT) mixture with addition of 1,3-beta-d-glucan (GLU, curdlan) was converted to be more elastic with maintaining mechanical properties of bioceramics and physicochemical properties of the surface [12,13]. Thus, it has huge potential in biomedical applications [14].

Raman spectroscopy and mapping have been reported as a successful approach for tissue characterisation and medical diagnosis [15]. They have been used in a variety of tissue origins including bones with their mineralisation ageing or bone composition studies [16,17,18,19]. However, Raman imaging in vivo suffers from several issues deriving from destructive and time-consumption restrictions of the technique. Size and shape of interesting specimens requires specific, sophisticated probes or expensive and bulky systems [15]. However, Raman mapping has been successfully demonstrated as an effective approach for tissue molecular composition determination. Coupled with multivariate analysis allows a quantitative and objective characterisation and tissues diagnosis [15].

The aim of tissue engineering is to produce biologically compatible three-dimensional substitutes for tissues. It is mostly beneficial in enhancing restricted regeneration according to the state of health, trauma or age [20,21]. There is an immense and permanent clinical need for developing technique providing fast, inexpensive and reliable information about biocompatibility of several newly discovered, potentially applicable implants. It is a scientific niche at the junction of rapidly developing tissue engineering and clinical surgery [22]. Currently, biomedical tissue engineering products follows well-established development path including preclinical, clinical and commercial stages, where a need for use of animals in research still exists [23]. During investigating bone implants, which requires surgery for implantation and euthanasia preceding examination of implant and surrounding tissue, there is no chance to avoid pain and distress. Attempts to limit the number of laboratory animals can be achieved in accordance with principles of the 3R (replacement, reduction and refinement) of the use of animals in research [24]. Both from disadvantages of in vivo application and renouncement of animal testing come clear conclusion that omitting one step by reduction can be beneficial from ethical and practical points of view. It is coherent with historically established law of the European Union, stating clearly that: “An experiment shall not be performed if another scientifically satisfactory method of obtaining the result sought, not entailing the use of an animal, is reasonably and practicably available”, thus developing intermediate method of bone formation evaluation by Raman spectroscopy is an opportunity and ethical obligation for novel composites development and investigation [25]. Following the replacement principle, meticulous investigation of specific multipotential cells in conditions close to physiological may confirm particular changes more quickly and cost-effectively involving Raman measurements and imaging.

The innovative approach presented in this work was performing in vitro cytocompatibility testing of biomaterial by means of Raman spectroscopy analysis using mesenchymal stem cells derived from two different tissues: adipose and bone marrow. We demonstrated that Raman spectroscopy is an effective approach for the analysis of biological samples and can be used in the biocompatibility studies of biomaterials applied in regenerative medicine, including bone tissue engineering, reducing the time-consuming in vivo animal testing only to the essential ones.

## 2. Results

### 2.1. Raman Spectroscopy

**Figure 1:**
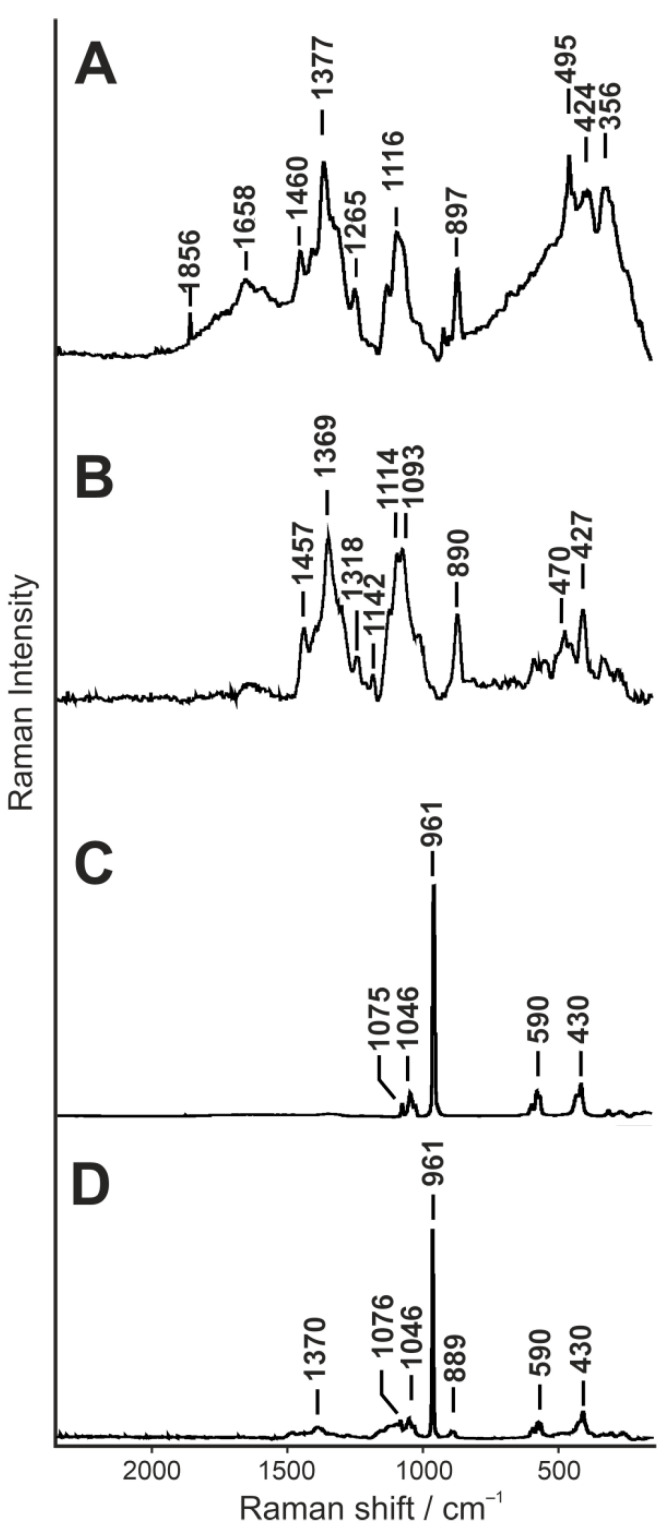
Representative Raman spectra of “raw” individual biomaterial components: CHIT powder (**A**); GLU powder (**B**); HA granules (**C**); and native biocomposite sample spectrum (**D**).

Figure 1A–C shows the Raman spectra of commercially available “raw” components used to produce biomaterial, recorded with 780 nm laser excitation. A representative spectrum acquired from native composite as a mixture of CHIT, GLU and HA is presented in Figure 1D.

In the spectrum of CHIT (Figure 1A) from sea krill, the bands at 897, 1265 and 1460 cm^−1^ are probably attributed to C-H out of/in plane and asymmetrical vibration. The band at 1116 cm^−1^ is attributed to C-O-C (ether) bond vibration, when the 1658 cm^−1^ indicates the C=O of Amide I and 1377 cm^−1^ is the C-N stretch. The region from 1600 to 1660 cm^−1^ with a band at 1658 cm^−1^ is connected with aromatic rings of polysaccharide [26]. The band at 495 cm^−1^ is assigned to CO-NH out-of-plane and C-CH_3_ in-plane bending. Pyranoid ring is characterised by presence of out-of-plane bending at 424 and 356 cm^−1^ [27,28].

β-1,3-d-glucan (curdlan) is a non-toxic and elasticity-increasing polymer due to the ability to form a firm, resilient gel in aqueous suspension [29]. This attribute was used to reduce the tendency for fragility of CHIT/HA material. The most significant and important for curdlan are bands at 427 cm^−1^ confirming presence of C-O-C β-glycosidic bonds as well as the band at 890 cm^−1^ associated with C-H deformation of β-glycosidic bond anomeric structure [30,31]. The absence of the band at 950 cm^−1^, assigned to α-1,3-glucan, proves that only one form of glucan is present in the sample. The polysaccharide nature is also confirmed by the band at 1369 cm^−1^ assigned to –CN stretching and presence of -CH_2_ and -COH groups, COC rings and ether bonds at 1093 and 1114 cm^−1^, respectively [13]. The band at 1318 cm^−1^ is assigned to –CH in-plane deformation, similarly to 1457 and 1142 cm^−1^, which are assigned to –CN stretching.

The hydroxyapatites’ (Figure 1C) high intensity band at 961 cm^−1^ corresponds to the symmetric stretching vibration (ν1) of the phosphate ion PO_4_^3−^. The bands at 1046 and 1075 cm^−1^ are typical for ν3 PO_4_^3−^ vibration, while those at 590 and 430 cm^−1^ for ν4 PO_4_^3−^ and ν2 PO_4_^3−^, respectively [30].

**Figure 2:**
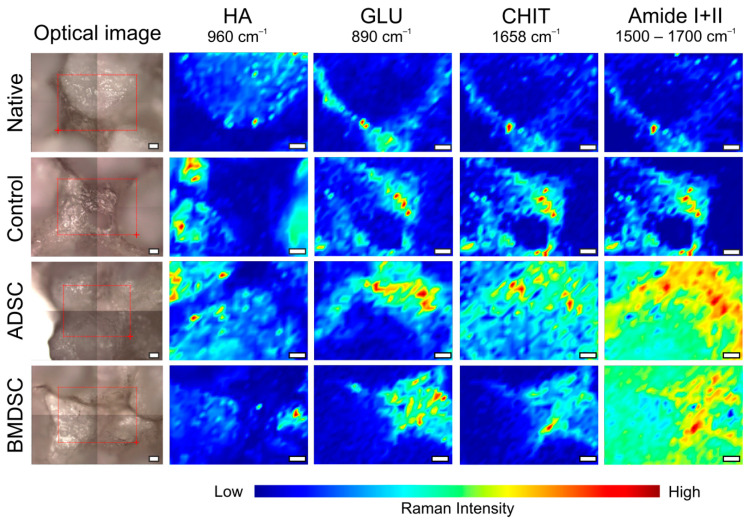
Raman microscopic images and chemical maps of scaffold compounds (HA, GLU and CHIT) and protein components (Amide I and II) distribution on the surface of samples untreated and treated with cells. White bars represent 50 µm.

In Figure 2, the visualisation shows distribution and intensity of signal of three basic biomaterials components in the x-y plane for each sample. Chemical distribution maps of 960, 890 and 1658 cm^−1^ bands corresponding to HA, GLU and CHIT, respectively, are shown. Differences in the distribution of the scaffold components in seeded and non-seeded samples indicate their transformation by growing cells. The residues of peptides and proteins assigned to Amides I and II are shown at map of distribution range from 1500 to 1700 cm^−1^. The intensity of Amides Raman signal in the sample shows that they are present in pockets created by area between hydroxyapatite granules as well as on the granules surface. The low intensity of Amides in control and native samples indicates relatively low protein accumulation during incubation, however the slightly higher intensity in control in this range than in native sample proves the adhesion of protein components derived from the cell medium. The much higher intensity of Amides band in ADSC and BMDSC samples suggests cell proliferation on the biomaterial surface. Presumably, these cells also produce protein components of bone ECM, such as collagen and osteocalcin, which indicates their differentiation towards osteoblast and thereby proper biocompatibility.

**Figure 3:**
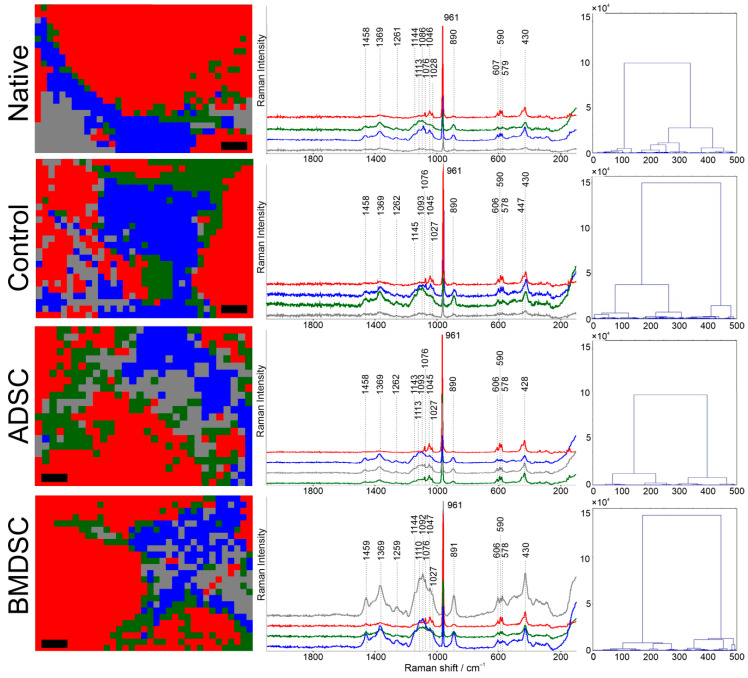
Hierarchical cluster analysis of four clusters with average spectra and dendrograms using D-value distance and Ward’s algorithm of native material, control sample, ADSC and BMDSC. Clustering shows groups of similar variables with the interpretation of similarity. Black bar represents 100 µm. The green spectrum represents cluster of GLU, the red spectrum represents cluster of HA, the blue spectrum represents cluster of CHIT and the grey spectrum represents cell remains on the surface of composite.

Hierarchical Cluster Analysis (HCA) calculated with D-values and Ward’s algorithm was applied to the Raman spectroscopic images shown in Figure 2. The results of subjected areas of clusters with dendrograms are shown in Figure 3. The Native, control, ADSC and BMDSC samples were clustered into four clusters. Three clusters represent the three raw components of biomaterial and the fourth cluster was assigned as cell remains.

ADSC and BMDSC maps show the difference comparing to native and control samples dividing into more clusters at the same level. The chemical distributions of clusters with assigned spectra were compared with acquired single components samples in Figure 1. Each of them shows spectra linked to signal band of hydroxyapatite with its broad presence in the samples. The native sample, untreated with cell medium and cells, shows averaged spectra of clusters with lower wavenumbers of Raman shift, compared to control, ADSCs and BMDSCs.

For each individual sample, four spectra are shown in Figure 3. One in every sample resembles the highest intensity of 961 cm^−1^ band assigned to hydroxyapatite. The two others might be related to mixed signals of two polysaccharides: CHIT and GLU. However, the characteristic for CHIT band at 1658 cm^−1^ is hardly noticed in each sample. The highest intensity of polysaccharide signal is found in BMDSC sample. It may also suggest deposition of carbohydrates constituents of ECM in this specimen.

### 2.2. Raman Imaging

**Figure 4:**
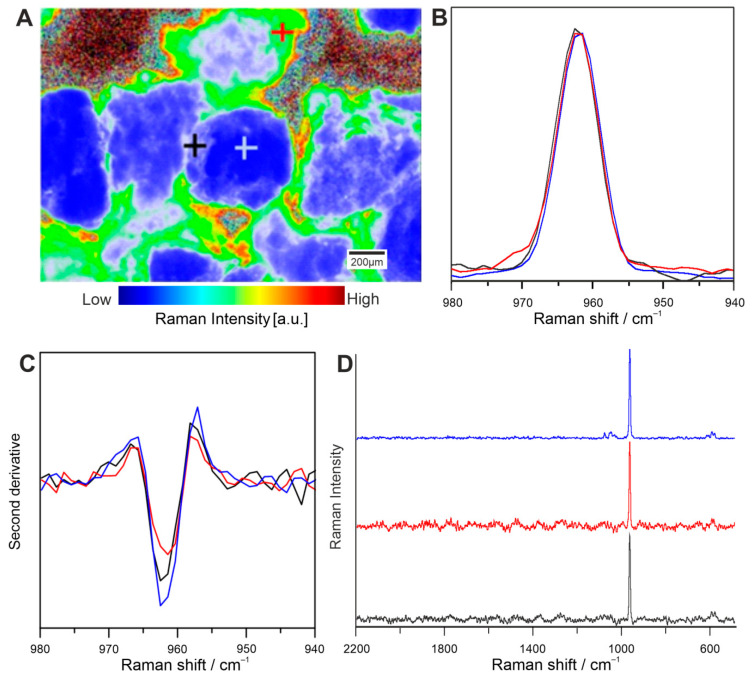
Raman imaging of ADSCs on the basis of: the second derivative of the 960/962 cm^−1^ ratio (**A**); the 940−980 cm^−1^ range spectra (**B**); their second derivatives in the 940−980 cm^−1^ range (**C**); and the whole spectra of chosen sites (**D**). The measurement spectra sites are marked with the crosses. The colours of the crosses (**A**) correspond to the colours of the lines (**B**–**D**).

The structural changes of HA can be detected by the changes appearing in the minimum of the second derivative of the spectra. Cells modify HA in different manner depending on the sample topography (Figure 4A)—they act diversely on the surface of HA granules and the boundary of HA granules and the organic matrix consisting of CHIT and GLU. Visibility of shifts is limited in spectra, but distinguishable in second derivative of range 940–980 cm^−1^ (Figure 4B–D). Slight shifts mean various HA structures. Such changes depend on the sampling place, indicating modifications of HA by ADSCs, such as phase transformation and/or crystallinity alterations. 

**Figure 5:**
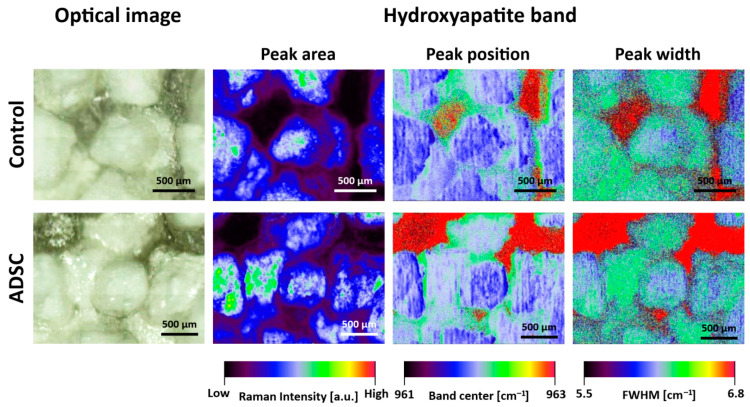
Raman hydroxyapatite imaging of control and ADSC sample. The analysis was carried out considering a Gaussian 960 cm^−1^ peak fitting (contributed to HA).

To compare HA distribution in control and ADSC sample, Raman imaging was performed. Figure 5 shows the maps of obtained HA peaks (area, width and position) with reference to the optical images.

Raman spectral imaging enabled generation of detailed chemical images based on HA composition and structure. Hydroxyapatite reconstruction in the initial phase is mostly expressed on the surface, in areas where HA granules are not present. the signal peak of HA at 963 cm^−1^ is assigned to more amorphous apatite, compared to highly crystallised HA used in biomaterial production, as shown in Figure 1. HA signal shift indicates that amorphous apatite was found alongside the one derived from composite, as shown by the peak position in the third column of Figure 5. Mineral, phosphate phase is distributed evenly as a thin layer, 4–13 µm depth, as reported in one of our previous works [32]. Thus, comparing to strong signal from concentrated granules of HA, phosphate signal originating from new HA layer appears in areas of lower intensity scale. Raman peak area related to peak intensity yields information of material distribution and concentration. Peak position ensures images of molecular structure and phase, and peak width provides data of crystallinity and phase. This analysis also indicates cell-induced HA structural changes in ADSC.

**Figure 6:**
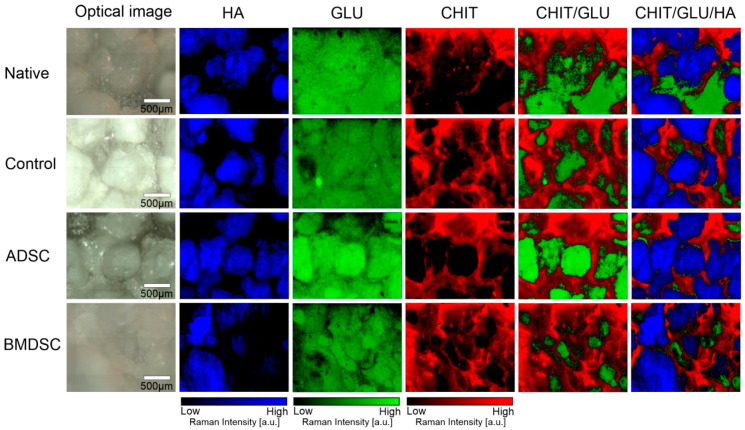
Raman imaging of chitosan/β-1,3-d-glucan/hydroxyapatite biomaterial samples with spatial distribution of components (CHIT, GLU and HA). The intensity of the colours does not reflect the percentage proportions between the particular components.

For detailed information about all compound distributions in all chitosan/β-1,3-d-glucan/hydroxyapatite biomaterial samples, surface and depth Raman imaging was carried out. This study allowed tracing not only the HA, CHIT and GLU separate arrangement, but also their mutual penetration. The results are presented in Figure 6.

Spatial distribution maps (Figure 6) show differences between the distribution of each component in the samples. Discrepancies occur mainly in compound of organic matrix (CHIT/GLU) arrangement and proportions. Surface maps indicated that in the ADSC and BMDSC samples the presence of the cells affect HA differently. In BMDSC sample, detected HA content is lower and HA granules are less pronounced in surface image of sample. This result may indicate increased mineralisation in ADSC compared to BMDSC sample, which affects the rate of formation and subsequent maturation of bone mineral.

**Figure 7:**
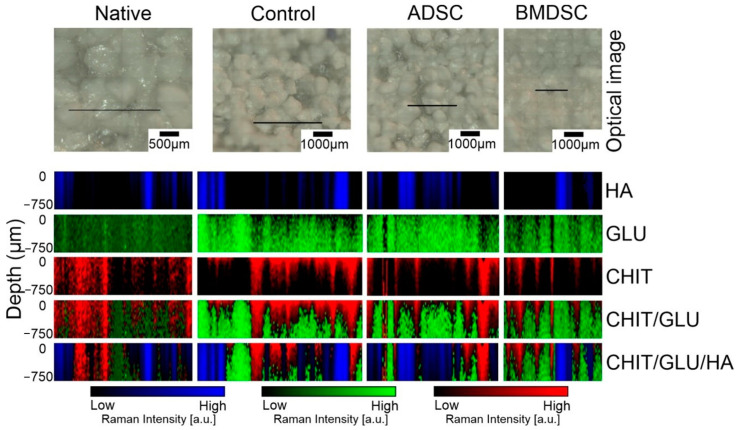
Confocal Raman microscopy imaging with depth-profiling in chitosan/β-1,3-d-glucan/hydroxyapatite biomaterials. Cross-section maps were deconvoluted using a standard component analysis procedure (DCLS Analysis, WiRE 4.4 Renishaw). The recorded spectra of the HA, GLU and CHIT were used as base spectra. Hydroxyapatite (HA) is marked blue, Chitosan (CHIT) in green and β-1,3-d-glucan (GLU) in red. Total Z-range of cross section was 750 μm of depth. The black line presented on the optical images indicates the region of interest in which the cross-section analysis was performed. The intensity of the colours does not reflect the percentage proportions between the particular components.

Figure 7 presents depth profiles of biomaterials. Confocal maps were made according to literature data, taking into account characteristic bands for each component. Individual visualisation of depth maps for hydroxyapatite (blue), β-1,3-d-glucan (green) and chitosan (red) are presented. Chit/Glu/HA biomaterial is in fact a heterogeneous mixture of compounds, which was also subjected to direct influence of cell medium and cell activity during incubation. Therefore, composition, arrangement and proportions are different, as biomaterial has undergone processes of formation and mineralisation of the bone. The results presented in Table 1 are supporting information to those available in the visual form in Figure 7. The blended colour images show penetration of components in the sample. The confocal resolution of each spectrum was set up to 50 μm in Z direction. The total Z-range from +50 to −700 μm was set up, where the zero level was the surface of the sample due to the roughness of the samples surface. CHIT was found mostly on the surface, penetrating in depth, mixing with GLU and more abundant in native sample. GLU signal appears in deeper layers of biomaterial widespread in all investigated samples. The relative contribution of individual components were calculated, and the results are presented in Table 1. Several maps (3–5) were analysed for each type of samples (native, control, ADSCs and BMDSCs) and the differences in the values of the relative contribution of each individual components (HA, GLU and CHIT) were not greater than 1% between the microRaman images of the samples of the same type, with the standard deviations not greater than 0.02.

## 3. Discussion

Biomaterials for bone tissue engineering applications are designed as a scaffold with macroporous and three-dimensional structure facilitating cell seeding. Their additional advantage is the possibility of seeding with patient cells in vitro prior to implantation, as a living autologous bone graft or tissue engineered product (TEP). The crucial factor for designing this kind of scaffold is evaluation for cytotoxicity according to ISO10993-5:2009 and confirmation of beneficial effect of medical application [33]. The mentioned standard focuses on the ionic surrounding microenvironment covering only part of interesting research features of bioceramics–cell medium–cells interactions. Currently, there are several techniques for evaluation and characterisation of composites among which can be mentioned magnetic resonance imaging (MRI) or Fluorescent labelling. Spectroscopic methods were proved to provide useful and unique information either prior to and after in vivo experiments because of their label-free, non-destructive and chemically specific properties during the investigation [30,34,35]. High-resolution Raman 2D images may be coupled with chemometric methods such as cluster analysis, revealing algorithm-resolved differences of acquired data.

In this report, the surface changes on tricomponent chitosan/β-1,3-d-glucan/hydroxyapatite biocomposite seeded with mesenchymal stem cells of different origin were evaluated. The purpose of this approach was to identify residues of stem cells, particularly presence of bone extracellular matrix (ECM). During the experiment period, the expression of proteins forming ECM on the scaffold surface by ADSCs and BMDSCs was affirmed, potentially of different phases of synthesis and bone maturation [36]. Identification of particular osteogenic formation protein markers allows indicating three phases of process: proliferation, ECM synthesis and ECM mineralisation [33]. Furthermore, Raman imaging confirmed the adsorption of proteins derived from cell culture medium to control biomaterial surface. All these findings are evidence of a high biocompatibility of the studied biocomposite.

Previous studies demonstrated that proper durability and macroporosity are the crucial features of good biocompatibility of bone implant [29]. In this case, biopolymers (CHIT and GLU) provide high osteoinductive properties, enhancing bone regeneration but with low mechanical stability [29]. However, this disadvantage was overcome by coupling them with calcium phosphate–hydroxyapatite–increasing scaffolds’ strength and resembling bone tissue. It is important for accelerating healing process to provide bone properties of graft. Tricomponent material becomes a scaffold for osteoblasts with facile adhesion due to the surface functional groups pattern. In designed TEPs, osteoblast-resembling stem cells can be involved in implantation, by culturing on graft in vitro, prior to surgical implantation. It reinforces biocompatibility of graft with concurrently maintaining its other advantages at initial phase of regeneration. Lining of niches surrounding stem cells is a process preceding remodelling of tissue, a natural cascade of rebuilding process.

Using HCA algorithms, grouped spectral images with averaged spectra were acquired. Each one of them has shown one distinct spectrum linked to signal band of hydroxyapatite with its broad presence in the samples. More detailed analysis of components interlacing is shown in Figure 6. HA, CHIT and GLU were imaged according to characteristic bands and imposed on the picture, visualising microscopic structures of mineral granules and filling biopolymer matrix. Mixture of components was also analysed in depth profile by confocal Raman analysis, as shown in Figure 7. Structures visible in Figure 7 were analysed in a two-dimensional manner as a linear map with in-depth steps of 50 and 750 µm in total. With this approach, it was possible to visualise the mixing of polymeric component of composite. Curdlan and chitosan were not mixed homogenously, but rather with layers, where curdlan covers composites surface, creating the surface with HA granules, indicating its important role in composite–cell interaction on the surface. Przekora et al. (2016) indicated important role of GLU on cell adhesion molecules in stimulating cell binding [29]. Signal intensity of GLU is relatively lower in ADSC and BMDSC samples compared to control, which may correspond with its involvement in facilitating interaction with stem cells, resulting in consumption of polysaccharide matrix. Furthermore, the lack of differences between native and control samples proves that the changes in the structure of the material occurs only under the influence of cell cultures and not only by incubation with the cell culture medium.

Well-established stages in development of new implantable materials are in vitro and in vivo tests. Both provide different information on, e.g., biocompatibility, inflammation or strength, with intention on further development of products. However, a surprisingly big part of reported data from animal experiments is barely cited in the following papers [37]. Ultimate sacrifice of animals is an indisputable necessity for commercial development of a product, but it seems to meet very little scientific interest expressed in citation count. It brings to a consideration where is the real point of balance between animal testing and profits achieved from experiments.

Due to a higher content of HA in ADSCs in relation to BMDSCs (Figure 2, Figure 6 and Figure 7), HA structure and crystallinity analysis was performed only for the ADSC specimen. Sample topography mainly comprised of hydroxyapatite granules with strong signal at 961 cm^−1^ was shown with imaging and chemometrics technique, combining optical image with chemical information. The intensity of signal in various points of magnified biomaterial is presented in Figure 4A, clearly exhibiting granules with interlacing polysaccharides. Spectra extracted from visually distinct points were analysed with second derivative algorithm revealing Raman shifts. They were identified as HA modification in terms of its phase transformation or crystallinity changes. Newly formed hydroxyapatite found in niches shows Raman shift to lower wavenumbers, which was confirmed in control and ADSC sample images (Figure 5) [38]. Distribution in control and ADSC samples was shown by peak area, compared to visible microscopic image. For differentiation of compound changes induced by surroundings peak width was introduced, indicating shift to higher wavenumbers, which was the result of highly crystalline structure of HA [39]. Proven formation of a new layer of HA in cell-seeded samples demonstrates surface biomineralisation resulting in a good bioactivity of the scaffold.

The current study focused only on Raman spectroscopy and imaging approach; however, to fully define the biocompatibility of the scaffold additional assays, studies should be performed, evaluating proliferation, gene expression and biochemical and histological characteristics of newly formed bone tissue. The disadvantage of Raman analysis is that it cannot specifically distinguish between cells that are just proliferating on the scaffolds compared to those that are undergoing complete osteogenesis. Extracellular bone matrix components deposition (e.g., collagen and osteocalcin) can be detected by gene expression (RT-PCR) and protein expression by immunohistochemistry [40,41]. Chit/Glu/HA scaffold was biologically evaluated in our previous published works with normal human foetal osteoblast cell line, where cytotoxicity and osteoblast proliferation were evaluated [29]. In another study, excellent spreading, focal adhesion formation, good proliferation and osteogenic-differentiation ability of ADSCs and BMDSCs were confirmed with confocal laser scanning microscopy (CLSM) [3]. Most recent work accurately analyses ECM with macro-ATR FT-IR imaging and CLSM, confirming production of collagen and osteocalcin in cell treated samples [42]. Mineralisation can be quantified by mineralisation assays or a calcium/phosphate-specific stain (e.g., Alizarine Red S and von Kossa); physicochemical methods should also be helpful, e.g., XMA (X-ray Motion) and XPS (X-ray photoelectron spectroscopy) analyses. Results from the Raman analysis should be cross-verified with the established methods for evaluating stem/progenitor cell activity and differentiation on biomaterial samples, which would be worth considering in our next research.

## 4. Materials and Methods

### 4.1. Biomaterial and Individual Compounds Used

The production method of chitosan/β-1,3-d-glucan/hydroxyapatite composite was developed and described previously by Przekora et al. (2014) [12]. Krill chitosan was obtained from Sea Fisheries Research Institute in Gdynia (Gdynia, Poland), β-1,3-d-glucan was commercially available curdlan from Wako Pure Chemicals Industries (Osaka, Japan) and hydroxyapatite granules were purchased from Chema Elektromet Rzeszow (Rzeszow, Poland). Briefly, biomaterial was prepared by mixing 1:1 two liquid phases: 4% (*w*/*v*) chitosan solution in acetic acid with 16% (*w*/*v*) curdlan suspension in distilled water. Then, 80% (*w*/*v*) HA granules were added to the obtained mixture and the homogenous blend was subjected to thermal gelation at 95 °C in a water bath, followed by neutralisation in sodium hydroxide (Avantor Performance Materials, Gliwice, Poland). Before the experiment, samples were sterilised by ethylene oxide.

### 4.2. Cell Culture

The study was performed using human adipose-derived mesenchymal stem cells and bone marrow-derived mesenchymal stem cells purchased from ATCC Cell Bank (ATCC-LGC Standards, Teddington, UK, Catalog No PCS-500-011™ and PCS-500-012™, respectively). Stem cells were cultured in Mesenchymal Stem Cell Basal Medium (ATCC-LGC Standards, Teddington, UK) supplemented with the components of Adipose-derived Mesenchymal Stem Cell Growth Kit Low Serum or Bone Marrow-Mesenchymal Stem Cell Growth Kit (ATCC-LGC Standards, Teddington, UK), respectively. Culture media were additionally supplemented with penicillin/streptomycin mixture (10 U/mL penicillin and 10 μg/mL streptomycin) purchased from Sigma-Aldrich Chemicals (Warsaw, Poland). Stem cells were cultured at 37 °C in a humidified atmosphere of 95% air and 5% CO_2_.

BMDSCs and ADSCs were seeded directly on 1 mm thick biomaterial samples (10 mm × 10 mm) that were pre-soaked in culture medium and placed in the wells of 24-well plate at a concentration of 5 × 10^4^ cells/sample. After 24 h incubation at 37 °C, the culture medium was replaced with osteogenic medium (Osteocyte Differentiation Tool, ATCC-LGC Standards, Teddington, UK). Every 3–4 days, half of the osteogenic medium was replaced with a fresh portion. Non-seeded biomaterial incubated in the culture media served as a test control and was referred as “Control” throughout the manuscript, whereas biomaterial seeded with ADSCs and BMDSCs was marked as “ADSC” and “BMDSC”, respectively. Control sample and biomaterials seeded with the cells were incubated for 20 days at 37 °C and afterwards fixed with 3.7% (*v*/*v*) paraformaldehyde (Sigma-Aldrich Chemicals, Warsaw, Poland) and subjected to further studies. Native (untreated) composite was stored in a Petri dish at 4 °C until use and was marked as “Native” sample.

### 4.3. Raman Imaging/Mapping

Raman imaging and mapping were performed using two different Raman spectrometers.

#### 4.3.1. Raman Mapping

Raman spectra were collected using a DXR Raman Microscope (Thermo Scientific, Waltham, MA, USA), with a 780-nm laser and an output power of 13 mW for obtaining signal with best Raman Intensity of mineral and organic compounds. The spectra were recorded in the spectral range of 200–2600 cm^−1^ using an operating spectral resolution of 4 cm^−1^ of Raman shift. A 25-pinhole aperture was used. The following settings for acquisition were used: exposure time of 6 s, number of exposures was 10 with 10× objective. Microscope was equipped with CCD Camera (Sentech, Ebina, Kanagawa, Japan) with 0.8 mega-pixel CCD sensor. Mapping consisted of 875 single measure points with step size of 25-µm. Samples were mapped with 10× objective and the use of autofocus at each map point because of height-diverted samples structure. To achieve good reproducibility of results, 2–3 microscopic images were acquired. Recorded optical images and chemical maps are presented in Figure 2 and Figure 3.

The spectra presented in Figure 1 were averaged from ten random measuring points of “raw” homogenous powders of CHIT, GLU and granules of HA. Additionally, in the same manner, ten single spectra of Native sample were recorded and averaged, as presented in Figure 1.

#### 4.3.2. Raman Imaging

Raman imaging and depth profiles were recorded with the inVia™ Confocal Raman Microscope (Renishaw, UK) equipped with EMCCD detection camera Newton 970 (Andor, Belfast, Northern Ireland, UK) and 20× long distance objective Olympus Plan N NA = 0.25 (Olympus, Shinjuku, Tokyo, Japan). A He-Ne laser (RL633 HeNe Laser, Renishaw, Wotton-under-Edge, Gloucestershire, UK) with wavelength of 633 nm and 25 mW power at the sample (or less) was used to collect the data. A 65-pinhole aperture was used. A subtraction of background signal derived from fluorescence and noise filtering was performed using WiRE 4.4 software (Renishaw, Wotton-under-Edge, Gloucestershire, UK). The spectra were recorded in the 500–2200 cm^−1^ spectral range, at 1 cm^−1^ spectral resolution (1200 lines/mm grating) (~400 × 300 points, 5 μm spatial resolution). Dept h profiles were as follows: step size of ×20 μm, y 20 μm and z 50 μm (z-range from +50 to −700 μm, where the zero level was the surface of the sample). At each point of acquisition, an exposure time of 0.4 s was used.

The confocal Raman images presented in Figure 7 were deconvoluted by a standard component analysis procedure (DCLS Analysis, WiRE 4.4 Renishaw, Wotton-under-Edge, Gloucestershire, UK). The recorded spectra of the HA, GLU, CHIT were used as base spectra. The reference spectra and all spectra of the map were corrected for the baseline before deconvolution. The deconvolution procedure was carried out taking into account the areas that cannot be fitted by base spectra set (Lack of Fit area) and without prenormalisation.

### 4.4. Data Analysis

All data processing was performed with OMNIC (ver. 8.2.0.387, Thermo Fisher Scientific, Madison, WI, USA) and CytoSpec (ver. 2.00.01, Berlin, Germany) software with baseline correction of spectra. The three spectra from each sample were collected and then averaged before analysis and comparison. For all maps, water vapour, thickness test and SNR quality tests were performed, prior to analysis. The hierarchical cluster analysis (HCA) was performed using the D-value distance (Pearson’s correlation coefficient) and Ward’s algorithm. As a result, dendrograms of classes were obtained, indicating further conduct of analysis. Then, four-cluster analysis was presented as a result, with spatial distribution and spectral data distinction in Figure 3.

## 5. Conclusions

The use of Raman mapping has been proven to be useful in characterisation of biomaterial samples and processed tissue engineered product. This microspectroscopic technique allowed identifying chemical changes resulting from incubation with the ADSCs and BMDSCs with micro-level spatial resolution and permitted detecting the local alterations in composition of biomaterial. Changes of HA crystallinity and its deposition on the surface of the tri-component chitosan/β-1,3-d-glucan/hydroxyapatite biomaterial together with detection of increased content of protein components in cell-seeded samples confirmed the bioactivity and biocompatibility of the designed scaffold, which promotes differentiation of somatic stem cells and ECM formation, thereby enabling proper osteogenesis process. Consequently, it can be stated that it has a great biomedical potential as an innovative biomaterial for the reconstruction and regeneration of bone tissue.

## Figures and Tables

**Table 1:** The relative contribution of the individual components to each sample.

	Native	Control	ADSCs	BMDSCs
HA	92.93%	71.13%	80.81%	73.44%
GLU	6.01%	28.85%	19.07%	26.53%
CHIT	1.06%	0.02%	0.12%	0.03%

## Data Availability

The data that support the findings of this study are available from the corresponding author upon reasonable request.

## Abbreviations

TEP	Tissue engineered product
ADSCs	Adipose-derived stem cells
BMDSCs	Bone marrow-derived stem cells
ECM	Extra cellular matrix
MSCs	Mesenchymal stem cells
HA	Hydroxyapatite
CHIT	Chitosan
GLU	β-1,3-d-glucan/curdlan
SNR	Signal-to-noise ratio
HCA	Hierarchical cluster analysis
MRI	Magnetic resonance imaging
XMA	X-ray Motion
XPS	X-ray photoelectron spectroscopy
CLSM	Confocal laser scanning microscope
